# Recent Advancement in Non-Enzymatic Electrochemical Detection of Lactate Based on Metal Nanomaterials: A Review

**DOI:** 10.3390/s25196194

**Published:** 2025-10-06

**Authors:** Chenxin Wang, Guanglei Li

**Affiliations:** 1 Xianyang Key Laboratory of Building Health Monitoring and Green Reinforcement, College of Civil Engineering, Shaanxi Polytechnic University, Xianyang 712000, China; ly2025012@163.com; 2 Interdisciplinary Research Center of Biology & Catalysis, School of Life Sciences, Northwestern Polytechnical University, Xi’an 710072, China; 3 State Key Laboratory of Transducer Technology, Shanghai Institute of Microsystem and Information Technology, Shanghai 200050, China

**Keywords:** non-enzymatic detection, metal nanomaterials, wearable lactate sensors, electrochemical, biofluids

## Abstract

Lactate is a vital biomarker for disease diagnosis and healthcare management. With the development of wearable sensors, by analyzing biofluids, such as sweat, saliva, and tears, it is possible to implement the in situ detection of lactate, which could provide clinical-grade data for early disease detection and personalized healthcare. Among them, non-enzymatic lactate electrochemical sensors (NELESs) are on the rise due to their quick response, are easily miniaturized, and have the ability to overcome the intrinsic disadvantages of enzymatic sensors. Compared with enzyme-based lactate sensors, NELESs could simplify the electrode preparation process, reduce the cost, and improve the sensing stability and service life. In this review, we introduce the significance of the real-time monitoring of lactate and highlight recent advances in wearable electrochemical sensors toward continuous lactate analysis in biofluids. In particular, metal nanomaterials have great potential in constructing NELESs due to their unique physical and chemical properties, which can be divided into four categories: bimetallic nanomaterials, transition metal chalcogenides (TMC), metal oxides, and layered double hydroxides. We discuss recent advances of these non-enzymatic lactate oxidation materials in detail, and provide some insights for the further development of NELESs through a comprehensive analysis.

## 1. Introduction

Under anaerobic conditions, glycolysis is the main pathway for organisms to obtain energy [1,2,3]. As a pivotal metabolite during anaerobic glycolysis, the levels of lactate in biofluids reflect the metabolic status of humans [4,5,6]. Therefore, lactate is commonly regarded as a signaling molecule in physiological and pathological processes [7,8]. For clinical diagnosis, the anomalous change in lactate levels could serve as an early warning signal for illnesses, for example, the process of tumor growth and proliferation produces large amounts of lactate, which is of great importance for the early diagnosis of cancer [9,10,11]. In addition, lactate levels can also be used to assess the mortality risk of patients. It follows that the real-time detection of lactate levels for patients could reduce the mortality rate [12,13]. As a consequence, it is of great significance to test the concentration levels of lactate in body fluids (such as blood, sweat, saliva, interstitial fluid (ISF), and tears). Over the past few decades, impressive advances have been made in disease diagnosis and monitoring by analyzing the lactate content [14,15].

Presently, lactate sensors can mainly be classified into two types: optical and electrical. Optical lactate sensors transmit the lactate concentration information by using optical signals such as absorbance, fluorescence, and chemiluminescence [16]. For instance, Zhang et al. [17] combined an AIE-active fluorophore (TPE-HPro) with L-lactate oxidase (LOx) to analyze the lactate concentration in aqueous solutions by fluorescence analysis with good sensitivity. In contrast, electrical sensors are easier to miniaturize and integrate [18,19,20,21,22,23]. Electrical sensors can generate electrical signals proportional to the concentration of lactate through electrochemical reactions, thereby achieving quantitative detection [24,25,26,27]. Jiang et al. [28] fabricated a wearable electrochemical sensor that was applied for the real-time measurement of lactate in epidermal sweat with a sensitivity of 40.6 μA mM^−1^ cm^−2^ and 1.9 μA mM^−1^ cm^−2^ in the range of 1–222 μM and 0.222–25 mM, respectively. Phumma et al. [29] utilized lactate oxidase, nanocellulose, and silver nanoparticles for the construction of a lactate electrochemical sensor, which could cover the diagnosis requirement of lactate concentration (12.5 mM) for muscle fatigue indication.

Although many researchers have made a lot of effort in the development of electrochemical lactate sensors, there were still some problems in the sensitivity and linear response range [30,31,32,33]. Furthermore, current lactate sensors are mainly based on an enzyme-based detection system. Despite their high sensitivity and excellent selectivity, enzyme-based lactate sensors still have some drawbacks such as high cost, poor stability, and complex production process. In addition, enzyme-based lactate sensors are easily affected by the environmental and experimental conditions including temperature, humidity, and pH values [34,35,36]. Hence, NELESs have attracted wide attention due to their outstanding stability, reasonable cost, simple manufacturing process, and good repeatability [37,38,39,40]. Nevertheless, the design of appropriate sensing materials is the key to solving this problem. Recently, the emergence of nanotechnology has provided an opportunity for the development of NELESs [41,42], of which metal nanomaterials have unique physical, electronic, and chemical properties, high surface area, and extraordinary catalytic activity [43]. These advantages provide metal nanomaterials with excellent sensing performances as well as non-enzymatic electrocatalytic activity for the electrochemical sensing of lactate.

In this review, the application of wearable electrochemical lactate sensors for different biological fluids is first introduced. Second, the advantages and disadvantages of enzyme-based and non-enzyme lactate sensors are summarized. Third, the current status of metal nanomaterials for the construction of enzyme-free lactate sensors are concluded and emphasized. Finally, the challenges and future development trends of NELESs are presented.

## 2. Biofluids Withdrawing Techniques of Wearable Lactate Sensors

**Figure 1:**
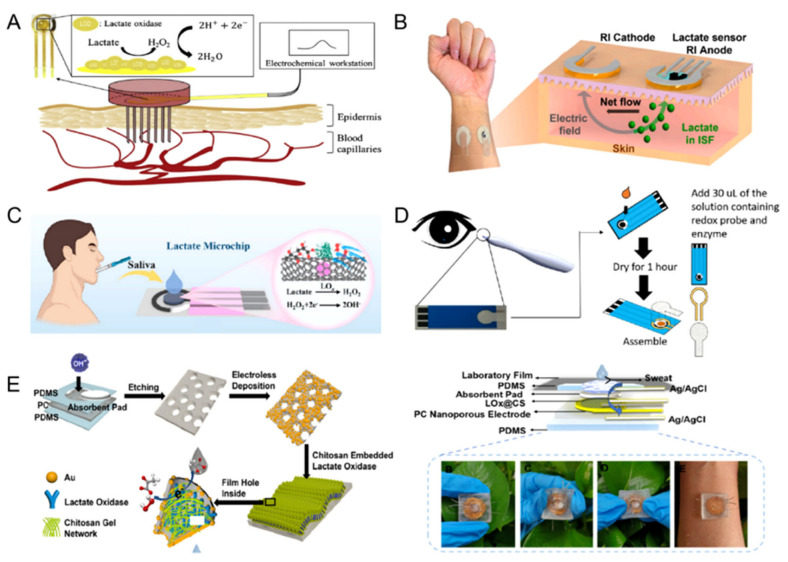
The application of an electrochemical lactate sensor for different biofluids. (**A**) Blood [46] Copyright 2020 Elsevier, (**B**) ISF [47] Copyright 2023 Elsevier, (**C**) saliva [48] Copyright 2024 American Chemical Society, (**D**) tears [49] Copyright 2018 Elsevier, (**E**) sweat [50] Copyright 2024 American Chemical Society.

In recent years, the integration of artificial intelligence and big data analysis has promoted the further development of wearable sensing technology that can analyze and summarize a large amount of physiological data collected from biofluids such as sweat, ISF, and tears, making it possible to formulate personalized health plans. These innovations have not only reduced the medical costs, but have also expanded the coverage of medical services [44]. As a common metabolite in the human body, lactate can be obtained from relevant biological fluids, such as blood, ISF, saliva, tears, and sweat, through invasive or non-invasive methods (Table 1 and Table 2, Figure 1) [45].

### 2.1. Blood

As the main biological fluid for lactate detection, the lactate concentration in healthy human blood ranges from 0.5 to 2 mM, which is measured via invasive methods [45,51]. Blood lactate, as a useful biomarker, is often used as a criterion for evaluating human diseases. When the lactate concentration is higher than 4 mM, it might indicate serious damage to the human body such as liver, kidney and cardiovascular dysfunction, and poor outcomes for shock patients. In acute cases, it might also lead to the growth of cancerous tumors [52]. At present, the detection of blood lactate mainly relies on expensive analytical equipment in hospitals via venous blood sampling. This process not only takes a long time, but also requires professional operators to obtain data and interpret the results. Although the existing portable sensors could monitor the concentration of lactate out of clinical conditions, most of them need to extract the blood from the body before testing. Huang et al. [53]. developed a flexible enzyme-free lactate sensing platform based on nickel oxide nanoparticles by inkjet printing. The LOD was 0.38 mM, and the linear sensitivity within a 0.6–2.2 mM range was 8.86 nA/mM/mm^2^, which could realize the detection of the lactate concentration in a patient’s blood plasma collected at the hospital.

To overcome the limitations, the development of wearable lactate sensors capable of real-time monitoring is of great significance. These could use microneedles to collect human blood in a minimally invasive manner and achieve in situ analysis and detection. Wang et al. [46] developed an electrochemical lactate sensor via three membrane electrodes. The membrane sensor attached to the microneedle array with lengths of 1000 or 1500 μm was able to realize the measurement of the lactate concentration in human blood by minimally invasive microneedle-based sampling to extract a small amount of body fluid (Figure 1A). The sensitivity of the membrane sensor based on a microneedle array for the measurement of lactate in the range from 5 to 30 mM was 0.337 μA mM^−1^. Compared with traditional centralized medical services, this sensor could directly obtain blood and plasma through a microneedle array, and only a trace amount of biofluid (~100 μL) was needed to achieve lactate detection, which could provide low-cost, time-saving and real-time health monitoring services for users.

### 2.2. Interstitial Fluid

ISF is the biological fluid that surrounds cells in the body, making up about three-quarters of the extracellular fluid and presenting in most dermis. The analytes in the ISF come from the blood through permeable and continuous capillaries and are mainly achieved through three ways: diffusion through the plasma membrane of endothelial cells, diffusion and/or advective transport between cells, and the vesicle transport of cells [51,54]. As a biological fluid containing rich biochemical indicators, ISF has considerable potential in obtaining health information of the human body. Lactate concentration for healthy person is in the range of 1–2 mM [45]. At present, there were two ways for wearable sensors to obtain ISF: invasive and non-invasive sensing technology. Among them, microneedles and reverse iontophoretic (RI) are the two most representative methods as they are minimally invasive on skin and have high stability. The microneedles used to obtain ISF can be solid, soft, and hollow, which can penetrate the dermis of the skin and avoid contact with the nerves due to their short enough length, making it a minimally invasive method. For solid microneedles, their tips could be functionalized with a sensing component to enable real-time monitoring through direct contact with ISF. The hollow microneedles could extract the ISF to the skin surface through vacuum and capillary action to achieve the detection of lactate concentration in the human body. Soft microneedles are often composed of hydrogels, which collect ISF through swelling [55].

Compared with microneedle-based sampling, RI is a completely non-invasive method that only acts on the skin surface, which could avoid potential surface biofouling caused by the need for skin penetration. Furthermore, there are successfully commercialized products (GlucoWatch) that have verified the feasibility of the RI system [47,55]. De la Paz et al. [47] presented a sensing technique that could achieve the non-invasive collection and detection of ISF lactate in15 min through a soft and adhesive wearable epidermal patch (Figure 1B). The patch was made up of medical tape, an RI system, a porous hydrogel reservoir (agarose and PVA),and an enzymatic amperometric lactate sensor. As a common technique, the application of the RI system was able to extract ISF via the formation of an electric field across the skin. This RI system consisted of an anode and cathode, which was separated from the skin through the porous hydrogel to prevent electrocution. When the electric field was applied, an electroosmotic flow was generated within the skin, which was able to make transport the ISF biomarker to the electrode. The lactate, as the anion, flowed toward the anode side of the RI system, where the sensor was capable of realizing the detection of lactate in human ISF by amperometry. The LOD for the developed lactate sensor was 0.15 mM with good reproducibility (RSD = 6%) and stability (~1 h and 35 min). This sensing patch only required a short RI process to collect tissue fluid, which reduced the damage to the skin surface caused by the application of an extended current. Meanwhile, the dynamic range of this wearable patch covered the lactate concentration range of healthy individuals at rest (1–2 mM), and its good reproducibility and stability indicate that this sensor has commercial potential and could meet the health monitoring requirements under sedentary conditions.

### 2.3. Saliva

The analytes in saliva are mainly derived from the capillaries that supply the salivary glands, so is a good correlation for the lactate content between saliva and capillary blood (r = 0.81) [51]. Therefore, saliva could be used as a non-invasive substitute for blood to reflect the health condition of the human body. In a healthy individual, the concentration of saliva lactate ranges from 0.11 mM to 0.56 mM [48]. Because saliva is abundant and easy to collect, it is of great significance to construct wearable saliva sensors when the real-time feedback of test results is required.

Zhang et al. [48] used MnFe PBA (Prussian Blue and its analogs) as a bimetal source because of the homogeneous distribution of Mn and Fe in its lattice frameworks, which can allow the transition metal nanoparticles to be uniformly embedded in the nitrogen-doped carbon nanotubes (N-CNTs) with melamine as a carbon/nitrogen co-precursor. The MnFe@N-CNT nanomaterial could serve as the signal probe. Its high degree of graphitization and low electrical resistance could enhance the electrocatalytic efficiency, which improves the sensitivity and detection limit of the sensor. The linear range of 0.1 to 3.7 mM could cover the fluctuation range of lactate concentration in saliva and satisfy the clinical requirement. When used as a screen-printing ink for the production of three-electrode microchips, the lactate concentration in patient saliva samples could be accurately measured within 30 s (Figure 1C). This represents a significant improvement over the 30 min required for lactate detection by high-performance liquid chromatography, which means that it can accurately measure rapidly changing lactate concentrations and has great potential for large-scale production. The development of this saliva-based biosensing microchip provides a powerful tool for the detection of periodontitis and also paves the way for the non-invasive detection of a wider range of biomarkers in saliva.

### 2.4. Tears

Lactate is one of the main bioanalytes in tears, usually derived from the corneal epithelium, from which lactate-proton exchange is transferred to the mesenchyma, further diffused to the endothelium, and into the tear film. The lactate concentration in tears is 1–5 mM, which is 4 to 10 times higher than that in the serum. Therefore, the non-invasive monitoring of lactate levels in tears could be used to assess certain health conditions in patients [45]. Currently, tear collection is mainly divided into two categories: non-stimulation (such as the capillary tube technique, test strip method) and stimulation. Lin et al. [49] successfully developed a reliable tear lactate test strip using a screen-printed sensor, double-sided tape, and filter paper. The insertion of this lactate test strip into the pen instrument and then touching the conjunctiva would provide an alternative method for those who do not like measuring lactate levels with contact lenses or needles. The sensor detected lactate from 0.39 to 16.60 mM, covering the clinically relevant lactate concentration range in tears (Figure 1D). This indicates that changes in tear lactate concentration caused by both local eye diseases (such as dry eye and hypoxia) and systemic diseases (such as sepsis, toxins, shock, anemia) can be captured. It was also not sensitive to the common interfering compounds in tears such as ascorbic acid, acetaminophen, and uric acid. In addition, this sensor could remain stable at 25 °C for eight weeks without signs of degradation. The excellent stability of this sensor at room temperature means that the replacement frequency and cost could be greatly reduced, which further ensures the reliability and consistency of the data.

### 2.5. Sweat

As one of the most popular body fluids for non-invasive continuous monitoring, the lactate content in sweat could be used as a biomarker for different pathological diseases. For healthy people, the concentration of lactate in sweat varies from 10 to 25 mM [45]. Compared with biological fluids such as blood, ISF, saliva, and tears, the collection of sweat is safer and more convenient. Sweat can be obtained directly from the surface of the skin by using sensor patches that do not produce an invasive impact on human skin. Therefore, the development of sweat-based wearable sensing platforms is of great significance for human health monitoring and has received extensive attention in recent years.

At present, there were two ways to collect human sweat: passive collection (such as physical exertion, hot shower, sauna) and active stimulation collection (such as drugs and electric field). Among them, physical exertion is the most common way to obtain sweat [55]. Wu et al. [50] developed a flexible wearable sensor for the simultaneous detection of body temperature and sweat lactate that could be attached to the volunteer’s arm through a layer of adhesive film and analyzed the sweat naturally produced by the volunteer during the exercise. Compared with other similar sensors, the nano-porous polycarbonate film could reduce resistance, accelerate catalytic reactions by utilizing nanoscale space, and simultaneously decrease substrate inhibition, thereby achieving a broader detection range and higher selectivity. Based on this, a wearable flexible sensor for real-time testing of sweat lactate was successfully built, which was able to be used for up to 13 days and had a linear coverage range of 0.01–35 mM with good selectivity (Figure 1E). Furthermore, the sensor could be used to estimate body temperature through a temperature-dependent transmembrane current. Long-term stability declared that the sensing performance attenuation was controllable, which ensured the reliability of the data. In addition, since the rate of enzymatic reaction was greatly affected by temperature, the dual function of the sensor could be used to adjust the effect of temperature on lactate detection and calibrate the data.

In summary, as a biological fluid secreted by organisms, the detection of human lactate concentration outside clinical conditions could be achieved by the real-time monitoring of biological fluids such as sweat, ISF, saliva, and tears. In recent years, with the significant development of electrochemical sensing technology, there has been considerable progress in the field of wearable devices (such as clothing, watches, and contact lenses) for the real-time monitoring of biofluids, which contain various biochemical components. These could be used to obtain biofluids non-invasively and conveniently to assess potential health conditions, which are of great significance to advance medical care and daily health.

## 3. Non-Enzymatic Lactate Sensing Material

For the sensing detection of lactate, it is mainly based on the enzyme-based detection system, which can be divided into two categories: lactate oxidase and lactate dehydrogenase. Natural enzymes can convert lactate to a substance with electrochemical activity, which is able to be oxidized and reduced to produce a current proportional to the concentration of lactate, in order to realize the sensing and detection of lactate [56,57,58].

However, due to the high cost, impressionable activity, and complex fixation method of natural enzymes, the promotion and use of lactate sensors have been restricted. In contrast, non-enzymatic sensors with a simple design and independence from biological components not only avoid oxygen interference, but are also more suitable for large-scale manufacturing [59]. Therefore, it is very important to design an electrode material that can detect lactate without enzyme sensing in promoting the development of lactate sensors. In recent years, the rise in nanotechnology has provided an opportunity for the development of electrochemical lactate sensors, in which metallic nanomaterials exhibited unique physical, chemical and electrical properties and have received extensive attention in the field of the enzyme-free detection of lactate. Specifically, they can be divided into four categories: bimetallic nanomaterials, TMC, metal oxides, and layered double hydroxides (Table 3).

### 3.1. Bimetallic Nanomaterials

Bimetallic nanomaterials are composed of two different metal components that can not only maintain the functional properties of each element, but also produce synergistic effects [71,72,73,74,75]. Compared with monometallic counterparts, the intricate interactions between the constituent metals of bimetallic nanomaterials provide them with unique properties that could improve the catalytic activity, selectivity, and stability. This synergy has driven the rapid development of bimetallic nanomaterials in the field of biosensing [76]. Taking Pd-M bimetallic systems as an example, the adsorption and activation ability of pure palladium materials for various molecules endowed them with excellent sensitivity and selectivity during the sensing process. However, the high cost of pure palladium materials affects its wide application, and its tendency to agglomeration, surface passivation, and deactivation during the electrochemical reaction process would lead to a decline in sensing performance. The introduction of a second metal, such as Cu, Ag and Ni, could not only reduce the amount of palladium, but also form a unique electronic structure and alloy effect to enhance the catalytic activity and electrochemical stability of the material [77,78]. At present, bimetallic nanomaterials have become ideal materials for the development of electrochemical sensors, and have gained wide attention in recent years.

Lu et al. [60] combined atomic layer deposition assistance techniques with hydrothermal strategies to prepare 3D-petal-like Ni_x_Co_y_ bimetallic 2D metal-organic frameworks (BMOFs) films on nickel foams (Ni_x_Co_y_ BMOF@Ni foams). In the resulting composite, the conductive nickel foam substrate could enhance the charge transport, while the NiCo BMOFs could provide a larger specific surface area and a richer active site than the mono-metal MOF membrane. For NiCo bimetallic sites, the presence of Co atoms favored the adsorption of lactate on them, making the initial dehydrogenation of lactate easier than the monometallic MOFs. With the increase in Co content, the current response became faster, and when the Co content was 5 times the Ni content, the current response to lactate was the best. The catalytic reaction process of lactic acid in composite materials is shown as follows:

NiCo_2_O_4_ + OH^−^ + H_2_O → NiO(OH) + 2Co(OH) + e^−^ (1)

CoO(OH) + OH → CoO_2_ + H_2_O + e^−^ (2)

Ni(III) + Co(IV) + lactate → Ni(II) + Co(II) + pyruvic acid(3)

**Figure 2:**
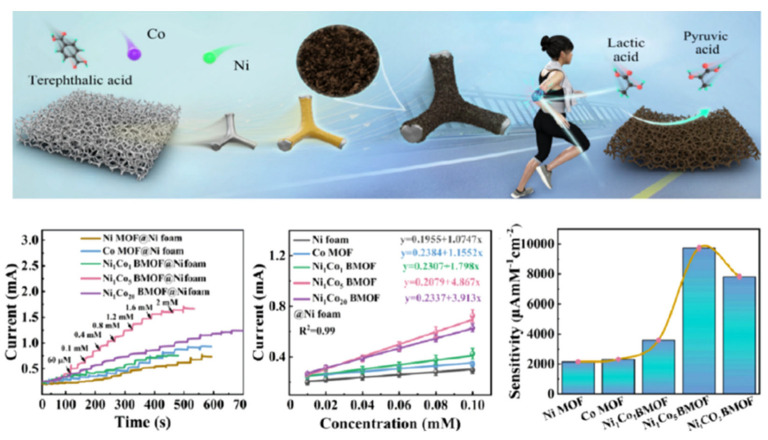
Illustration of the application of NixCoy BMOF@Ni foams in lactate sensing [60]. Copyright 2024 American Chemical Society.

Therefore, the NiCo BMOF@Ni foam, in a relatively low concentration of lactate ranging from 0.01 to 2.2 mM, showed ultra-high sensitivity (9030 μA mM^−1^ cm^−2^) and a low limit detection of 0.16 μM, which successfully achieved the enzyme-free selective detection of lactate (Figure 2).

### 3.2. TMC

The first TMC compound was discovered by Linus Pauling in 1923, and by the end of the 1960s, about 60 TMC compounds had been reported [79,80]. In 2004, graphene was discovered by mechanical exfoliation, and its unique physical and chemical properties received attention for layered materials. The rapid development of related research has also promoted the development of TMC [81]. As an inorganic graphite analogue, TMC is composed of transition metal atoms sandwiched between monatomic sulfide layers such as sulfur, selenium, and telluride; its typical chemical formula is MX_2_ (M = transition metals, X = S, Se, Te) [82,83,84]. The hierarchical structure of TMC is maintained by strong molecular bonds within the layer and weak van der Waals forces between the layers. This structure provides TMC with a large specific surface area, an adjustable band gap [85], good biocompatibility [86,87], and excellent electrical properties [88], which makes it a very attractive biosensing material [89]. On the one hand, the large specific surface area of TMC could provide more binding sites for the target biological molecules. On the other hand, because of the smaller atomic number of congeneric elements and the larger band gap, the sulfur element usually has a stronger binding effect on the valence electrons. Since the appropriate band gap is one of the key factors affecting the function of the sensor, transition metal chalcogenides are increasingly used in the field of biosensing [61,62,90].

**Figure 3:**
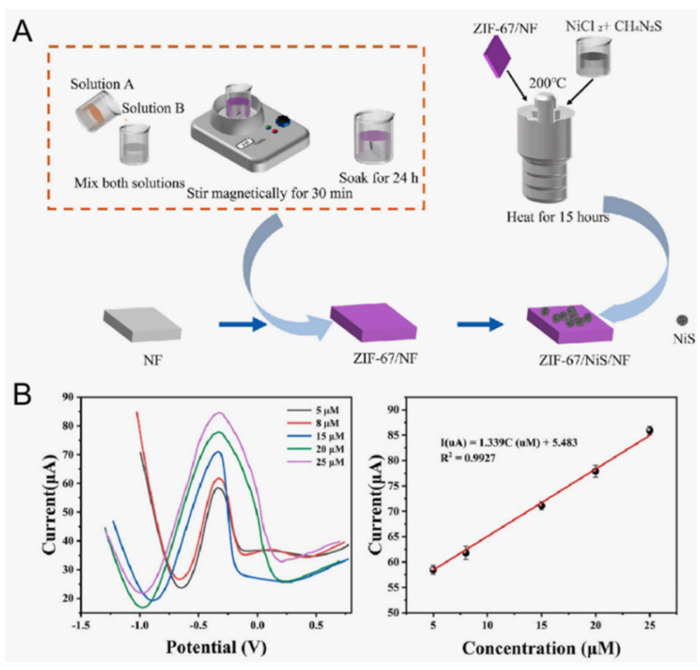
(**A**) Scheme of the synthesis method for the ZIF-67/NiS composite electrode. (**B**) The sensing performance of the ZIF-67/NiS electrode for lactate [63]. Copyright 2024 Elsevier.

Tao et al. [63] prepared a ZIF-67/NiS composite electrode on a nickel foam (NF) matrix through the hydrothermal method (Figure 3). The small band gap of transition metal sulfides and the low electronegativity of S were conducive to the transition of electrons, which gave them unique electrical properties. During the electrochemical detection process, NiS was prone to Ni^2+^/Ni^3+^ conversion. Ni^2+^ would first be converted into Ni^3+^, and then the formed Ni^3+^ was able to effectively oxidize lactate molecules to pyruvate through the double electron transfer process to achieve the enzyme-free detection of lactate. Its detection sensitivity in alkaline solution was 1.34 μA μM^−1^ cm^−2^, and the detection limit was 0.8 μM. The detection limit of this sensing system was much lower than the lowest physiological lactic acid concentration in all biological fluids, which indicates that it could capture the minute lactate concentration fluctuation signals in the human body. Meanwhile, its high sensitivity made the sensing signals easy to read, which is of great significance for the development of miniaturized and high-precision wearable lactate sensors.

Xiao et al. [64]. combined screen-printed electrodes (SPEs) with nanocomposites by electrodeposition to construct an enzyme-free lactate sensor based on molybdenum disulfide nanosheets modified by gold-platinum bimetallic nanoparticles. For one thing, the unique layered structure and good electrical conductivity of molybdenum disulfide nanosheets provided the high sensitivity of this sensor. For another, the gold-platinum bimetallic nanoparticles were capable of replacing the biological enzymes to achieve the oxidation of lactate (Lactate + O_2_ + MoS_2_-AuPt → pyruvic acid + H_2_O_2_). Hence, the sensor could achieve the efficient enzyme-free detection of lactate with a low response time (less than 15 s) and detection limit (0.33 μM), which was successfully applied to the detection of lactate in human sweat. The quick response speed of this sensing system and its detection limit was much lower than the lowest physiological lactic acid concentration in sweat, which indicates that it could capture the earliest and weakest pathological signals and has great potential in clinical applications.

### 3.3. Metal Oxides

Compared with nitrides, carbides, and phosphates, metal oxides are easy to prepare and have better stability at ambient conditions and in alkaline media [91,92]. In addition, the multivalent states of transition metals might be able to enhance the electrocatalytic activity of the sensing electrode, especially for nickel (Ni) and copper (Cu) as well as their oxides [65,93]. As a consequence, metal oxides have become promising electrode materials and have been welcomed in the field of enzyme-free electrochemical sensing [66,94,95]. Take copper oxide as an example. Copper, as a naturally abundant element, is second only to silver in its electrical conductivity. It has good biocompatibility and can be widely used. Copper oxide, as its stable oxide, is composed of copper atoms and four oxygen atoms. It is a p-type semiconductor with a narrow band gap (1.2 eV), excellent electrical conductivity, and stability. As a low-cost catalytic material, CuO has been widely used in electrochemical sensing [96,97,98].

**Figure 4:**
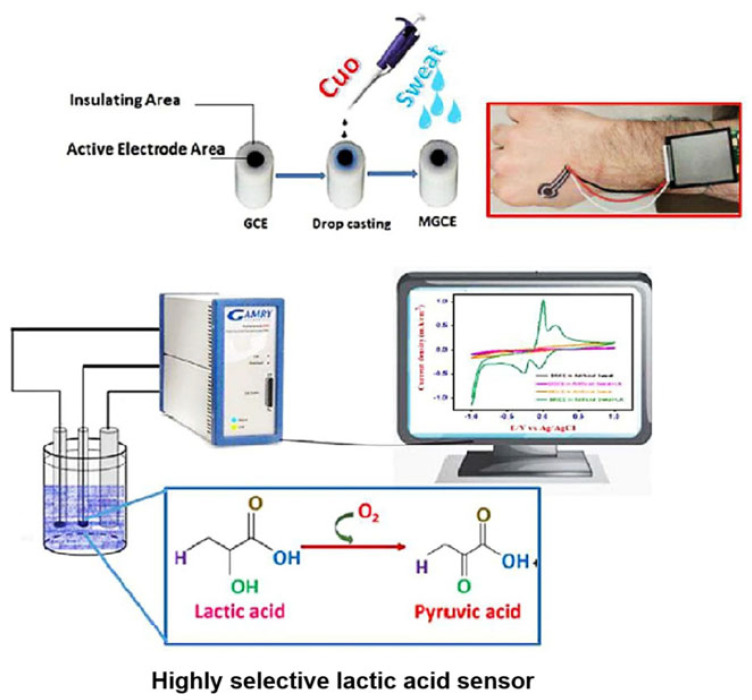
The electrochemical sensing mechanism of the CuO electrode for lactate [67]. Copyright 2023 Elsevier.

Sajna et al. [67] prepared copper oxide nanoparticles by aqueous precipitation. As a non-toxic transition metal oxide, copper oxide has high stability and catalytic activity, which can successfully achieve the non-enzymatic detection of lactate. When electrochemically exposed with CuO, the lactate was electrochemically oxidized to pyruvate and hydrogen peroxide in the presence of oxygen. Its detection covered and exceeded the range of normal lactate concentration in sweat without physical activity (0.05–40 mM). As an important non-invasive method, the utilization of CuO nanoparticles provides an excellent route for the non-enzymatic specific detection of LA biomolecules (Figure 4).

Tsou et al. [68] designed a flexible non-enzymatic lactate sensor by using a working electrode that was printed with 30 μL NiO_x_ ink and 30 μL NiO mixed with 4 μL Nafion ink. In aqueous solution, the hydrogen ions of hydrophilic sulfonic acid groups (-SO_3_H) in Nafion films form H_3_O^+^ molecules with water molecules, and the presence of negatively charged SO_3_^−^ ions could make the films block negatively charged interfering substances. Therefore, as an ion exchange membrane, Nafion membranes could provide better anti-interference for sensing materials. Compared with the pure Nafion membrane, the application of the NiO_x_-Nafion nanocomposite layer could have both the function of an anti-interference layer and reaction layer. The pure NiO_x_ layer at the bottom is mainly involved in the redox reaction, converting lactic acid to pyruvate (Ni^3+^ + lactate → Ni^2+^ + pyruvate). The electrode material based on this design scheme could enhance the sensing current. As a consequence, this sensor exhibited a high sensitivity of 20.56 nA/mM/mm^2^, low LOD of 0.27 mM, and excellent anti-interference ability of more than 95%, which could meet the criteria for human lactate measurement. In addition, the plasma lactate detection results of the sensor in clinical trials showed a strong linear correlation (0.959) with lactate levels measured by colorimetry used in hospitals, which means that the sensor has clinical application potential.

### 3.4. Layered Double Hydroxides

As a host–guest laminated material, layered double hydroxides (LDHs) are composed of electrostatic attraction between the positively charged host layer and anionic guest molecules. Its structure is similar to brucite, consisting of a metal hydroxy-octahedron M(OH)_6_ with a common edge [99,100]. The chemical formula of LDHs has been described as [M_1−x_^2+^M_x_^3+^(OH)_2_]^x+^[A^n−^]_x/n_·mH_2_O, where transition metals such as Co, Ni, Cu, Cr, Fe, and Mn could be used as divalent (M^2+^) and trivalent metal cations (M^3+^) [101,102,103,104,105]. At the same time, A^n−^ acts as an n-valent exchangeable anion that could be replaced by multifarious anions (inorganic and organic) and metal complexes such as ferrocene derivatives, nitrate ions (NO_3_^−^), carbonate ions (CO_3_^2−^), and metal porphyrins (FeTSPP) [106,107]. The wide range of compositionability of metal cations and interlayer anions in the hydroxide layers provides a highly adjustable chemical composition and structure as well as a variety of physicochemical advantages for LDHs, which give them excellent electrocatalytic performance and biocompatibility, and so have received extensive attention in the field of enzyme-free biosensing [108,109,110].

Wang et al. [69] prepared a NiCo LDH with an inverse spinel structure by using co-based ZIF-67 as a template. Since Co and Ni exist in multiple valence states and can simultaneously act as solid-state redox pairs, they could further provide synergistic effects in electrocatalytic reactions. At the same time, it has a layered structure with high porosity and a high electrochemically active surface area, which is conducive to electron transfer and could accelerate lactate oxidation to obtain excellent non-enzymatic lactate sensing performance. The catalytic reaction of ZIF-67NiCo LDH for lactate is shown as follows:

Ni(OH)_2_ + OH ⟷ NiOOH + H_2_O + e^−^ (4)

NiOOH + Lactate ⟷ Ni(OH)_2_ + pyruvate (5)

Co(OH)_2_ + OH ⟷ CoOOH + H_2_O + e^−^ (6)

CoOOH + Lactate ⟷ Co(OH)_2_ + pyruvate (7)

This electrode material achieved ultra-high sensitivity of 83.98 μA mM^−1^ cm^−2^ in a concentration range from 2 to 26 mM. Furthermore, the sensor for lactate measurement based on the ZIF-67-derived NiCo LDH displayed excellent long-term stability and high anti-interference performance, which could be used for high-accuracy non-enzymatic lactate monitoring in human sweat.

**Figure 5:**
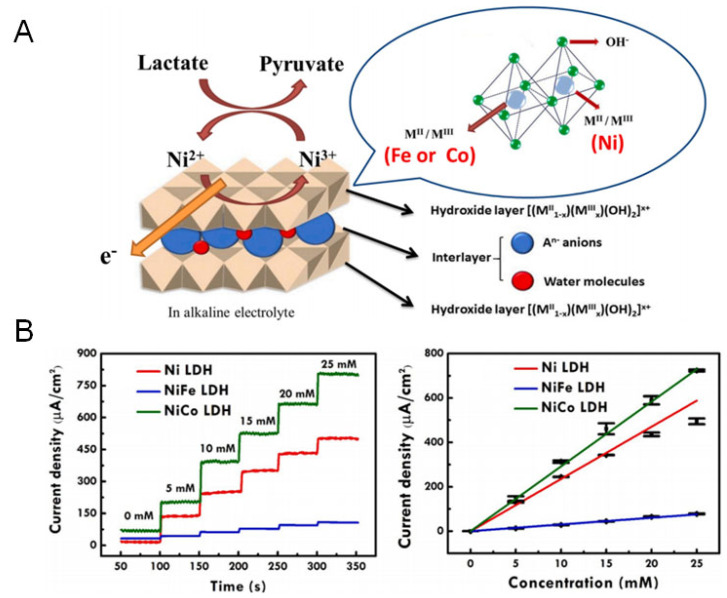
(**A**) Scheme of the oxidation reaction for lactate on bimetallic Ni-based LDHs. (**B**) The sensing performance of the NiCo LDH electrode for lactate [70]. Copyright 2021 Elsevier.

Wu et al. [70] combined various transition metals (Co and Fe) with Ni-based LDHs and used them as sensing materials for the construction of enzyme-free electrochemical lactate sensors. The introduction of the transition metal Co into Ni-based LDHs could improve the adsorption of OH in alkaline electrolyte and promote the oxidation of lactate. Therefore, the sensitivity of NiCo LDH modified screen-printed carbon electrodes (SPCEs) was 30.59 ± 0.34 μA mM^−1^ cm^−2^ in the lactate concentration range of 5 to 25 mM. This was higher than that of the Ni LDH (23.51 ± 0.45 μA mM^−1^ cm^−2^) and NiFe LDH (3.03 ± 0.06 μA mM^−1^ cm^−2^) modified SPCEs. The sensing electrodes exhibited high accuracy, excellent long-term stability, and could be reused many times, which provide unlimited possibilities for the construction of various enzyme-free lactate electrochemical sensors (Figure 5).

## 4. Conclusions and Prospects

For clinical diagnostics, the accurate and real-time monitoring of lactate in human biofluids is urgent. Based on this, electrochemical lactate sensors with low detection limits, high sensitivity, and reasonable cost have received much more attention. In this review, a detailed insight into the latest advances in metal nanomaterial-based enzyme-free lactate sensors was discussed from four aspects including bimetal, transition metal chalcogenides, metal oxides, and layered double hydroxides.

Although the development and research of metal nanomaterials have made great progress in the construction of non-enzymatic electrochemical lactate sensors, there is still spacious room for their further growth. At present, the sensor detection of biological molecules is mostly carried out under experimental conditions through specific substrates and solutions, while its practical application in the human body would involve more complex situations. Unlike the high selectivity and sensitivity of natural enzymes to specific substrates, metal nanomaterials could effectively respond to various substrates under different biochemical conditions, so they might cause false signals when detecting biological fluids containing various trace elements, electrolytes, inflammatory factors, proteins, and so on. Therefore, the selectivity of metal nanomaterials in the construction of non-enzymatic lactate biosensing needs to be carefully considered in the design to avoid wrong signals. In recent years, the development of nanotechnology has provided assistance in the enzyme-free selective detection of biological molecules. On the one hand, screening could be achieved by designing nanomaterials with specific pore sizes; on the other hand, selective detection could be realized by introducing specific functional groups. Furthermore, for electrochemical reactions, the maximum response to the target substrate and the minimum response to interfering substances could be achieved by optimizing the working potential. Meanwhile, the development of artificial intelligence can also provide assistance for the optimization of potential.

It was worth noting that the use of sensors with a single function is limited due to the multiple analytes contained in body fluids. Therefore, it is of great significance to develop a sensor that can simultaneously and multiplex screen target biomarkers on the basis of maintaining the independent and selective operation of a single sensor. This would not only greatly reduce the cost, but also provide convenience for users, and is an important direction in the development of future sensors.

In addition, the recent use and development of nanostructured materials has promoted the advancement of biofluid detection technology and improved the sensing performance of electrochemical sensors. With the continuous progress of biosensing technology, wearable devices that could achieve clinical detection and treatment through real-time and reliable health monitoring has become a future goal. Due to the high elasticity of human skin and the long-term surface attachment of wearable devices, sensing electrodes with good biocompatibility and remarkable operability in bending and stretching states play a key role in the design of wearable sensors.

In summary, lactate, as an important biochemical parameter, exists in various biological fluids. At present, numerous lactate electrochemical sensors have been designed based on the different biofluids, but the selective detection of lactate mainly depends on biological enzymes. However, due to the fact that the activity of biological enzymes is easily disturbed by external environments (such as temperature and pH value) as well as their high cost, the large-scale production of these sensors is not conducive. Hence, the development of non-enzymatic lactate sensing materials has received extensive attention. Among them, metal nanomaterials have often been used to construct NELESs due to their unique physicochemical properties and designable structures. These can mainly be classified into four categories: bimetallic nanomaterials, TMC, metal oxides, and layered double hydroxides. Although certain achievements have been made, further research is still needed in terms of selectivity, flexibility, and multi-functionality.

## Figures and Tables

**Table 1:** Recent work regarding lactate detection in blood, ISF, saliva, tears, and sweat.

Electrolyte	Sensitivity	Linear Range	Limit of Detection (LOD)	Response Time	Stability	Reproducibility (Relative Standard Deviation, RSD)	Application (Biological Samples)	Ref
Blood plasma	0.337 μA mM^−1^	5–30 mM	-	15 s	8 h	-	Blood plasma	[46]
Phosphate buffer solutions (PBS, 0.1 M, pH 7.4)	-	0–5 mM	0.15 mM	-	~1 h and 35 min	6%	ISF	[47]
10 mM PBS (pH 6.5) containing 0.1 M KCl	85.17 μA/(mM cm^2^)	0.1–3.7 mM	-	30 s	2 weeks	6.41%	Saliva	[48]
Simulated tear fluid	-	0.39–16.60 mM	-	-	8 weeks at 25 °C	-	Tears	[49]
PBS (0.1 M, pH 7.4)	0.0824 μA·mM^−1^	0.01–35 mM	0.144 μM	-	13 days	3.34%	Sweat	[50]

**Table 2:** Comparison of several parameters in blood, ISF, saliva, sweat, tears, and urine.

Biofluid	Withdrawing Techniques	Components	Lactate Concentration	Potential Interferents	Detection Challenges	Can Require Stimulation	Ref
Blood	Venous blood sampling Microneedles	Plasma, blood cells	0.5–2 mM	Uric acid, glucose, Na^+^, K^+^, Ca^+^, urea, ascorbic acid	Difficult to collect via non-invasive methods	No	[45,46,51,52,53]
ISF	Microneedles RI system	Similar to plasma	1–2 mM	Similar to blood	Most of ISF is gelatinous, which is difficult to collect	No	[45,47,51,54,55]
Saliva	Non-invasive direct collection	Dilute secretion (99% water), contains many different enzymes, electrolytes and other components	0.11–0.56 mM	Similar to blood	Interference from daily dietary secretions and the accumulation of oral bacteria	No	[42,45,48,51,54]
Sweat	Passive collection (such as physical exertion, hot shower, sauna) Active stimulation collection (such as drugs and electric field)	Perspiration contains a large amount of water and a small amount of electrolyte, glucose, lactate, and other substances.	10–25 mM	Similar to Blood	High evaporability of sweat and contamination on the skin surface (such as dust, oil)	Yes	[42,45,50,51,54,55]
Tears	Non-stimulation (such as capillary tube technique, test strip method) Physical and chemical stimulation	Tears contain many elements such as lysozyme, immunoglobulin, sugar, and inorganic salts	1–5 mM	Similar to Blood	Difficult to collect and high requirements on the safety of sensors	No	[45,49,51,54]

**Table 3:** Summary of NELESs.

Classification	Sensing Material	Electrolyte	Sensitivity	Linear Range	LOD	Stability	Reproducibility (RSD)	Application	Ref.
Bimetallic nanomaterials	Ni_x_Co_y_ BMOF@Ni foams	0.1 M NaOH	9030 μA mM^−1^ cm^−2^	0.01−2.2 mM	0.16 μM	-	-	-	[60]
TMC	NiF/HS-NiS	1.0 M KOH	0.655 μA μM^−1^ cm^−2^	0.5−88.5 μM	0.023 μM	5000 s	2.3%	Urine	[61]
	NiS-NC@NiS-MS	1.0 M KOH	0.39 μA μM^−1^	0.5−85.5 μM	0.5 μM	5000 s	2.3%	Urine	[62]
	ZIF-67/NiS composite	1.0 M KOH	1.34 μA μM^−1^ cm^−2^	5 μM–25 μM	0.8 μM	30 days	2.2%	-	[63]
	MoS_2_-AuPt	0.01 M PBS (pH 7.4)	-	0.005–3 mM	0.33 μM	30 days	0.8%	Sweat	[64]
Metal oxides	Porous NiO	0.1 M NaOH	62.35 μA mM^−1^ cm^−2^	-	27 μM	-	-	-	[65]
	NiO nanoparticles	0.1 M NaOH–KCl	1.564 μA mM^−1^	0.1–5 mM	0.03 mM	-	-	-	[66]
	CuO nanoparticles	Artificial Sweat (pH 7.4)	14.47 mA mM^−1^ cm^−2^	0.05–2.5 mM	0.027 mM	-	1.12%	Sweat	[67]
	NiO_x_/NiO_x_-Nafion	0.01 M PBS (pH 7.4)	20.56 nA mM^−1^ mm^−2^	0.5–4 mM	0.27 mM	-	-	Blood plasma	[68]
Layered double hydroxides	ZIF-67 derived NiCo LDH	0.1 M NaOH	83.98 μA mM^−1^ cm^−2^	2–26 mM	0.399 mM	28 days	-	Sweat	[69]
	NiCo LDH	0.1 M NaOH	30.59 μA mM^−1^ cm^−2^	5–25 mM	0.53 mM	28 days	-	-	[70]

## Data Availability

No new data were created or analyzed in this study. Data sharing was not applicable in this study.

## Abbreviations

The following abbreviations are used in this manuscript:

NELESs	Non-enzymatic lactate electrochemical sensors
ISF	Interstitial fluid
LOD	Limit of detection
RSD	Relative standard deviation
PBS	Phosphate buffer solution
RI	Reverse iontophoretic
N-CNTs	Nitrogen-doped carbon nanotubes
LOx	Lactate oxidase
TMC	Transition metal chalcogenides
SPEs	Screen-printed electrodes
LDHs	Layered double hydroxides
